# Pathway Profiling and Rational Trial Design for Studies in Advanced Stage Cervical Carcinoma: A Review and a Perspective

**DOI:** 10.5402/2011/403098

**Published:** 2011-07-06

**Authors:** Susy M. E. Scholl, Gemma Kenter, Christian Kurzeder, Philippe Beuzeboc

**Affiliations:** ^1^Département d'Oncologie, Institut Curie, 75005 Paris, France; ^2^Centrum Gynaecologische Oncologie Amsterdam, AMC, NKI-AVL, and VUmc, 1007 MB, Amsterdam, The Netherlands; ^3^Department of Gynacologic Oncology & Gynaecology, University Hospital of Essen, Henricistrasse 92, 45136 Essen, Germany

## Abstract

Multiple genetic abnormalities will have occurred in advanced cervical cancer and multiple targeting is likely to be needed to control tumor growth. To date, dominant therapeutic targets under scrutiny for cervical cancer treatment have been EGFR pathway and angiogenesis inhibition as well as anti-HPV vaccines. The potentially most effective targets to be blocked may be downstream from the membrane receptor or at the level of the nucleus. Alterations of the pathways involved in DNA repair and in checkpoint activations, as well as the specific site of HPV genome integration, appear worth assessing. For genetic mutational analysis, complete exon sequencing may become the norm in the future but at this stage frequent mutations (that matter) can be verified by PCR analysis. A precise documentation of relevant alterations of a large spectrum of protein biomarkers can be carried out by reverse phase protein array (RPPA) or by multiplex analysis. Clinical decision-making on the drug(s) of choice as a function of the biological alteration will need input from bio-informatics platforms as well as novel statistical designs. Endpoints are yet to be defined such as the loss (or reappearance) of a predictive biomarker. Single or dual targeting needs to be explored first in relevant preclinical animal and in xenograft models prior to clinical deployment.

## 1. Inequality in Cervical Cancer Incidence and Mortality in Europe

In economically developed countries with adequate screening practices the incidence and mortality rates of cervical cancer have been stable over the last 7 years ([1] and B Monk (IGCS meeting, Prague 2010)) with a low mortality-to-incidence ratio (< than 0.3)). The mortality remains; however, high (ratio of 0.8) in populations with inadequate or absent screening practices [2]. In Europe, the age standardised incidence of invasive cervical cancers (all stages) was estimated for 2004 to be 10 per 100.000 women-years. These statistics are from the “earlier” 15 member states of the European Union (EU), situated in West and South Europe. According to recent statistics, there remains a manifest disparity in incidence and mortality rates of cervical cancer across the extended EU of 27 member states. Incidence rates as high as 17 per 100 000 women-years have been quoted for the ten new member states that joined the EU in 2004 and are located predominantly in Central and Eastern Europe. In Bulgaria and Romania [3], the two most recent member states, that acceded to the EU in 2007, rates were still higher with an age-standardised incidence recorded in 2004 of respectively 20, and 22 per 100 000. The mortality of cervical cancer in Romania is approximately twelve times higher compared to that of Finland, the country in Europe with the lowest cervical cancer burden at present [4].

## 2. State of the Art in the Management of Advanced Stage Cervical Carcinoma

While early stage localized cervical cancer can most often be treated by surgical resection only and has excellent survival statistics, the presence of lymph node or parametrial involvement calls for chemotherapy and radiotherapy, most often in association.

Major international groups such as the GOG (gynaecological oncology group) and the EORTC (European organisation for research and treatment of cancer) as well as many national groups have been instrumental in the development of the present standard of care. 

### 2.1. Evidence—Based Present Standard of Care for Stages IB2-III Disease

Concurrent chemoradiation with a platinum-based agent is the recommended standard of care for locally advanced cervical cancer of stages IB2 to III. This standard has been developed through a successive series of clinical trials culminating in the GOG 120 trial published by Rose et al. [5] in the NEJM in 1999. Further trials, adding 5-FU (GOG 165) or Hemoglobin support (GOG 191), did not further improve on this standard. More recently, the addition of gemcitabine to the standard chemo-radiation showed a small but significant benefit in survival (*P* = 0.022); however, it cannot be ruled out that the higher efficacy may be confounded by two additional cycles of adjuvant chemotherapy. Currently, a significantly increased hematologic and digestive tract toxicity blocks broad implementation of such a protocol (Dueñas-González A et al. JCO 2009 [A 5507]).

### 2.2. Outcome of Stage 1B2 to III Cervical Cancer Patients Who Are Not in Complete Remission following Best Standard of Care

In a retrospective review of patients treated between 2003 and 2006 at Institut Gustave-Roussy, the outcome for patients treated for cervical cancer was poor for those who, following initial chemo-radiation, had not achieved a complete histological remission in either the hysterectomy specimen or in their lymph nodes. Patients died at a median time interval of 11 months after surgery (range 3–21 months) and further surgery did not improve the outcome [6]. Similar data have been published by a group from Montpellier [7].

For patients who fail first-line therapy, subsequent courses of chemotherapy are even less effective. Despite treatment with cisplatin or cisplatin and paclitaxel, the median survival time [8] of stage IV or of recurrent disease patients in GOG protocol 169 was approximately 8-9 months. There was no difference between groups, and only 15–20 percent of patients remained alive at 18–20-month followup. The palliative care is complex and carries a heavy toll on health resources but foremost on patients and on their families.

### 2.3. Ongoing Trials to Improve Standard of Care of Stage Ib–III Cervical Cancer

Ongoing, recently started or soon to start phase III trials with a “classical design” ask questions on the benefit of enhancing the radio chemotherapy sequence. Of note are the following.

- A presently ongoing phase III protocol (GOG 219) that compares standard radio chemotherapy with the addition or not of TPZ (tirapazamine), a radio-enhancing agent, testing the effect of increased free radical formation at the tumour site.
- In a randomized trial within the EORTC Gynae-Oncology Group (EORTC 55994) the outcome of neo-adjuvant chemotherapy followed by radical surgery is being compared to primary chemo-radiation for stages Ib2–IIb. Currently over 500 patients have been randomized.
- The OUTBACK trial (ANZGOG) evaluating the effect of 4 additional cycles of adjuvant paclitaxel and carboplatin (IB2-IV) to standard radio chemotherapy.
- The INTERLACE trial (NCRI) is in development and aims to test the addition of neoadjuvant chemotherapy with paclitaxel and carboplatin prior to chemo-radiation.

To our knowledge there are presently no ongoing large phase 3 trials in cervical cancer which involve-so-called “targeted” biological reagents with the exception of bevacizumab (NSC 704865). Two phase 2 trials are presently ongoing in stage Ib–III cervical cancers, involving cetuximab in association with radiation and cisplatin. In France a multicentre randomized phase 2 trial (CETUXICOL *n* = 76) evaluating cetuximab (CTX) in first-line treatment in association with chemoradiotherapy has been initiated by Institut Curie, in interaction with the national French group Fédégyn. (The following French Cancer Centres participated in this trial: Bergonié, BORDEAUX; P Strauss STRASBOURG; J Godinot REIMS; Val d'Aurelle, MONTPELLIER; F Baclesse, CAEN; P Calmette, MARSEILLE; A Vautrin, NANCY; R Gauducheau, NANTES; GF Leclerq, DIJON; C Regaud, TOULOUSE, and Institut Curie (PARIS & ST CLOUD).) This protocol recruited its first patient in March 2009 and concluded patient recrual in June 2011. The primary objective of this trial is to assess whether the addition of cetuximab to standard therapy will impact on DFS at 18 months. Secondary objectives are the analyses of genetic and protein biomarkers which may predict response to therapy. Patients have not been screened upfront as regards to pathway activation or downstream mutations but biological assessments are ongoing. Hopefully we shall be able to discern biological patterns that are associated with response or progression in cervical cancers in the near future which will allow us to plan new trials accordingly. 

### 2.4. Standard of Care and Ongoing Trials for Patients with Very Advanced (Stage IV) or with Recurrent Disease

Present options for the management of stage IV disease consist of palliative therapy including chemo-radiation to the pelvis if technically feasible, which is preceded or followed by more chemotherapy. For practical reasons these two settings are frequently regrouped in clinical trial protocols. There are several shortcomings: firstly, in the recurrent situation there is blood vessel fibrosis as a result of previous radiotherapy, decreasing drug availability at the tumour site. Secondly, these tumours are inherently resistant to radiotherapy and not highly sensitive to chemotherapy and newer treatment strategies are desirable.

A series of ongoing or recently closed phase 1 and phase 2 trials in late stage disease which explore chemotherapy in association with targeted therapies or targeted therapies alone were discussed at the recent IGCS meeting in Prague, October 2010 (Table 1). A number of targeted agents either as single drugs or in association with another biological agent or chemotherapy are being assessed in solid tumours not otherwise specified and include angiogenesis, Braf, mtor, akt, mek, notch inhibitors, among others (http://www.cancer.gov/cancertopics/types/cervical).

## 3. Trials in Advanced Cervical Cancers Based on Tumour Biology

### 3.1. Where Is the Cervical Tumour's Achilles Heel?

Advanced cervical cancer is a relatively rare disease in developed countries. Cervical cancer biopsies, albeit of easy access, are not available in large numbers. At this stage the three dominant targets under scrutiny for innovative cervical cancer treatments are the EGFR pathway, angiogenesis inhibition, and anti-HPV vaccines. This may rapidly change as knowledge of interactions between pathways develops.

There is to our knowledge no published data on representative series with in-depth exploration of EGFR copy numbers, protein expression, on polymorphisms, activating mutations or truncations. There is presently no information on how any of these alterations could relate to response to EGFR inhibitors. Assessing the activity (or lack of activity) of an antibody targeting the extracellular domain of the receptor may not be informative in the absence of knowledge on downstream molecular activating mutations, such as ras/raf, PI3K, and activations of mek, akt, mTor, and so forth. Over expression of EGFR, frequently assessed only by IHC, is commonly seen in 2/3 of cervical tumour samples. Retrospective data on a consecutive series with long term followup from the Charité Hospital in Berlin [9] did detect correlations with outcome following radiotherapy and/or surgery but their data suggested that EGFR (HER1) overexpression was associated with a *favourable* outcome (*P* = 0.006) Concomitant overexpression of HER2 or HER3, favourite heterodimeric binding partners for HER1 was interestingly highly significantly associated with a poor prognosis (*P* = 0.006). Data on phosphorylation status was not available. None of these patients treated in the 90s had received an anti-EGFR inhibitor, and virtually none of these patients had received any chemotherapy at all. In a more recent publication, Noordhuis et al. [10] evaluate the prognostic impact of an activated EGFR pathway as measured by IHC testing of phosphorylated EGFR (19,7%), AKT (4,1%), PTEN (34,1%), and ERK (29,2%) on prognosis. The study is based on pretreatment samples of a population of 375 stage Ib-IVa cervical cancer patients treated with standard chemoradiation. The authors report that membrane EGFR (35,3%) staining was inversely correlated with PTEN staining. Membrane staining of EGFR correlated with cytoplasmic staining of pEGFR and both were independent predictors of *poor response* to chemoradiotherapy. Positive staining was defined as 2-3 positive staining in at least 10% of tumour cells. No data on hetero dimeric binding partners was reported and there has been no significant exploration on genetic amplifications of EGFR, PI3K and so forth. to our knowledge.

Tumour growth and invasion is dependent on blood supply which makes angiogenesis inhibition one of the cornerstones of cancer treatment. But angiogenesis may not be an important target if the angiogenesis *inducing mechanism* is effectively targeted as suggested by dual targeting approaches discussed earlier. The vascular endothelial growth factor (VEGF) and receptor family is upregulated in conditions of hypoxia, and in particular by hypoxia inducible factor 1. HIF1*α* in turn is stimulated by HPVs as well as by activated membrane tyrosine kinase receptors such as EGFR and IGF1R. HIF1*α* has also been shown to be induced by apoptosis. There is furthermore ample evidence that angiogenesis is induced by members of the PIK3/mTOR pathway [11–14] as well as by oncogenic papillomavirus genes E6 and E7 [15, 16] themselves. Single and double targeting strategies should be helpful to discern the need to target angiogenesis specifically, beyond the targeting of the immediate angiogenesis causing mechanism.

More than 90% of cervical cancers bear one or more high risk HPV types and have activation of viral E6 and E7 genes and consequent loss of RB [17] and p53 [18] function, leading to a loss of the capability to undergo apoptosis as well as to enhanced telomerase activity [19]. HPVs thus encode the two tumour-specific oncoproteins E6 and E7 that act synergistically to maintain the malignant phenotype [20–23]. pRB degradation by E7 prevents its binding to E2F which in turn regulates expression of genes involved in progression to the S phase of cell cycle, such as cyclin D and CDK2 genes. Polo-like kinases (Plks) are involved in the assembly and dynamics of the mitotic spindle apparatus and in the activation and inactivation of CDK/cyclin complexes. Inhibitors of Plks are being developed by pharmaceutical companies and may prove of interest in cervical cancer treatment [24, 25]. However, tumorigenesis can be induced by either overexpression or downregulation of Plks, suggesting that the level of Plk has to be tightly regulated, reflecting its critical role in mitotic progression. Excessive Plk1 will override the mitotic checkpoint amplify the centrosome abnormally, and chromosomes will segregate without proper alignment or unequally. Insufficient Plk1 will also lead to mitotic delay and improper separation of chromosomes [26–28]. In both scenarios, aneuploidy and tumors will occur and genetic alterations in the EGFR-PI3K pathway may be secondary events. 

On the positive side, the HPV proteins E6 and E7 can act as tumor antigens and elicit a favourable immune response in which specific T cells play a critical role in the control and elimination of the HPV infection. The virus-specific interferon-*γ*-producing CD4^+^ cells and CD8^+^ cytotoxic T lymphocytes (CTLs) are able to recognise the oncoproteins E6 and E7 and contribute to the viral elimination [29–31]. However, in case of an uncontrolled persistent infection with a high-risk type HPV, the expression of the viral oncoproteins E6 and E7 contributes to the development of cervical (pre)malignancies. Apparently, the spontaneous HPV-specific T cell response failed in these patients and there is no or only a negligible expansion or activation of the proper HPV16-specific CD4^+^ and CD8^+^ T cells [32–34]. Combining current treatment strategies such as (chemo)radiotherapy or chemotherapy singly in association with immunotherapy could offer a novel approach for such patients. The rationale for exploring these combined treatments is the positive immune system stimulation demonstrated by several chemotherapeutic agents. In addition, preclinical data have shown promising results with therapeutic vaccines. The safety and immunogenicity of a synthetic long peptide E6/E7 HPV 16 vaccine was established in a phase 1/II trial in women with recurrent cervix carcinoma [35]. Similar data have been achieved with a viral vector based vaccine and [36]. Clinical trials are currently ongoing in preinvasive CIN3 as well as in cervical cancer patients using either peptide-based vaccines or mutated E6 and E7 gene sequences in a poxviral vector. Furthermore, the combined immunostimulatory effect of chemotherapy with long peptide HPV vaccination is being tested in an ongoing clinical trial.

### 3.2. Thoughts on Therapeutic Prospects

#### 3.2.1. Epithelial Growth Factor Receptor Tyrosine Kinase

EGFR is overexpressed in more than half of all cervical cancers and its phosphorylated form is a dominant feature in 20% [10], suggesting that receptor EGFR blockage remains a promising target. Strategies towards EGFR inhibition in advanced and recurrent cervical cancers by either anti-EGFR antibodies (cetuximab) or by small molecule tyrosine kinase inhibitors (gefitinib, erlotinib, and lapatinib) are ongoing but have not yet been conclusive. Reasons for the absence of a major clinical breakthrough may be multiple. The absence of the molecular target in nonscreened patients, the presence of downstream activating mutations, the high degree of resistance to standard therapy in the advanced setting, and the redundancy in pathway activations may limit clinical responses to distinct molecular phenotypes. Data from head and neck (H&N) cancer biopsies suggested that high EGFR gene copy number (Fish+) was associated with a significantly poorer outcome (review in expert opinion: [37]). It is uncertain whether results on EGFR function in H&N cancer can be extrapolated to cervical cancer; however, both are squamous type carcinomas and both have an HPV-related aetiology, suggesting that there might be activation of common molecular features. The response pattern to EGFR inhibitors in H&N tumours also remains controversial since low to moderate IHC staining for EGFR expression [37] was shown to correlate better to treatment than high EGFR expression. It was hypothesized that the dosage of anti-EGFR inhibitors may not have saturated the receptor in high expressing tumours and further studies are warranted. Cetuximab, an antibody targeting membrane EGFR, was shown efficient in improving the long-term survival of patients in association with radiotherapy [38, 39] and is approved as an active drug in H&N tumours.

EGFR inhibition also remains a privileged target of investigations in cervical tumours, based on the link between EGFR activation and resistance to radiation and platinum-based chemotherapy [10]. While EGFR is activated by phosphorylation in 20% of cervical cancers [10], no mutations have been demonstrated so far to our knowledge, which is very different from lung cancer which harbours close to 20% (559/2880) mutations in the EGFR kinase domain [40]. EGFR variant III is a predominant feature of glioblastomas [41] and has been associated with radiation resistance and hypoxia tolerance to our knowledge [42]. No data is available on EGFR variant III in cervical cancer. The prediction of a response to EGFR targeted therapy in cervical cancer remains a matter of debate since no precise molecular studies are available. Consequently, no decisive results have been achieved in early trials of nonselected patients with late stage disease (GOG 76-DD). Solid predictive markers of response are needed [43]. Other membrane receptors may be activated in the presence of HPV infection such as hepatocyte growth factor and c-met [44] or insulin-like growth factor receptors [45]. 

Different drugs that target EGFR show more or less clinical activity depending on the state of the molecular target. Erlotinib performed best in lung cancers with high levels of EGFR activation and activating mutations in the kinase domain [46]. There is no evidence of activating mutations in the EGFR in cervical cancer to our knowledge. Lapatinib in breast cancer was active predominantly in the presence of phosphorylated EGFR and HER2 while Herceptin is active in breast cancers with increased copy number of HER2 (Fish). A nuclear translocalization mode of EGFR has recently been reported in a variety of cancer cell types [47], and the effect of cetuximab on nuclear EGFR has been investigated with variable results [48, 49]. Acquired resistance to cetuximab has been linked to increased levels of nuclear EGFR in lung cancer [48] while lapatinib supposedly inhibits nuclear translocation of both EGFR and HER2 [50]. Nuclear EGFR, beyond its kinase function, is thought to have functions in gene regulation and in protein-protein interactions [47]. It is supposedly implicated in a number of processes, including DNA repair and resistance to DNA-damaging radiation and alkylating anticancer agents. EGFR nuclear entry was suggested to be blocked by lapatinib [50] and the Src family kinase inhibitor, dasatinib [48]. Celecoxib has been shown to inhibit radiation-induced nuclear EGFR transport [51]. The ability of nuclear EGFR to enhance expression of iNOS [52] and in particular Aurora Kinase [53] is challenging and worth further assessments. Equally, EGFR inhibition together with apoptosis inducing agents may have synergistic antitumor effects [54]. These observations provide a rationale for selecting novel combination treatments that overcome nuclear EGFR-mediated therapeutic resistance, but these mechanisms need to be documented in cervical cancer biopsies and tested in preclinical xenograft studies.

#### 3.2.2. PI3Kinase/PTEN and Ras/Raf Pathways

While preclinical studies on cell lines suggested an added benefit of combining cetuximab with cisplatin and radiotherapy, as measured by decreased MAPK and AKT phosphorylation, results were *independent of the level of EGFR expression*. In some, but not all, cervical cancer cell lines a benefit was seen in combining cetuximab with an anti-TKI, an anti-MEK1/2, or even with trastuzumab suggesting that targeting EGFR alone may not be enough. 

Recent laboratory evidence using colorectal cancer cell lines suggested that expression status of EGFR ligands (amphiregulin and epiregulin mRNAs) might need to be evaluated as dynamic predictors of response in KRAS wild-type (WT) patients receiving cetuximab-(CTX-) based therapy. The transcriptional control of the epithelial-to-mesenchymal transition (EMT) by keratins, focal adhesion signalling (integrins), and EMT-inducing cytokines such as TGF*β* as well as the upregulation of the epithelial markers E-cadherin has also been thought to be predictive and worth further assessments [55]. Other downstream effectors, such as Cox-2 overexpression [56], warrant further exploration. Germ-line polymorphisms for EGF, EGFR, Cox2, Cyclin D1, and FCGR2A may also be significant predictive markers for treatment response in lung cancer [57], but data from patients with cervical tumours treated with EGFR inhibitors is critically needed. The detection of a constitutively activating polymorphism at codon 655 of HER2 has been described [58] to be specifically associated with advanced carcinoma of the uterine cervix. The same authors also showed that patients bearing the CCND1 A/A and A/G genotypes (of cyclin D1) displayed a 1.811-fold increased risk of cervical cancer (95% CI = 1.150–2.852, *P* = 0.0098).

#### 3.2.3. Angiogenesis

Cervical tumours are frequently highly vascular, bleeding spontaneously, and on minor contact, and the addition of antiangiogenic agents has consequently been tested in a series of clinical trials. Bevacizumab in association with first-line radiochemotherapy in untreated locally advanced cervical cancer patients was well tolerated and is currently being evaluated in several phase 2 trials designed to test the therapeutic efficacy in association with chemo-radiation (RTOG 0417) as well as in combination with topotecan and cisplatin as first-line therapy for recurrent or persistent cervical cancer (GSK 107278). A phase III trial is ongoing testing the value of bevacizumab in association with two chemotherapy doublets, (NSC 708465), and the results are eagerly awaited.

#### 3.2.4. Angiogenesis and EGFR Inhibition Combined

Based on the assumption that cervical cancer is heavily dependent on EGFR activation and on angiogenesis (in turn caused by EGFR activation and HPV infection as well as by the hypoxia-induced VEGF), a phase II trial was designed to compare a multitarget TKI and antivascular agent (pazopanib) and an oral inhibitor of activated HER1 and HER2 (lapatinib). Each agent was given alone or in combination in advanced or recurrent cervical cancer patients, not previously selected on the basis of molecular assessments. Pazopanib proved overall superior to lapatinib. Pazopanib improved PFS (hazard ratio (HR), 0.66; 90% CI, 0.48 to 0.91; *P* = 0.013) and OS (HR, 0.67; 90% CI, 0.46 to 0.99; *P* = 0.045), and, interestingly, a negative interaction between agents was suggested in the combination arm, but complete dosage of both drugs could not be achieved due to toxicity [59]. A similar negative correlation through the addition of cetuximab to bevacizumab in metastatic colorectal carcinoma resulted in a significantly shorter progression-free survival when both agents were combined [60]. Since angiogenesis induction is enhanced by both inappropriate HPV effects on p53 and RB as well as by EGFR pathway activation it might be useful to assess countering HPV activity and EGFR pathway activation in preclinical trials.

#### 3.2.5. Immunological Targets

In preclinical experiments, a prerequisite for successful application of therapeutic vaccines hinges on effective induction of effector T-cell responses. Recently, a highly immunogenic synthetic long peptide (SLP) vaccine, consisting of long overlapping peptides of the E6 and E7 oncogenic proteins of HPV, has been developed and tested clinically. The vaccine elicited strong and broad HPV16-specific CD4^+^ and CD8^+^ T-cell responses in patients with cervical cancer without toxicity beyond grade 2 and was well tolerated by patients. The immune response did; however, not yet result in measurable clinical beneficial effects, and the therapy should be explored further [35, 61]. Markers of the integrity of an immune response that may result in clinical benefit are critically needed. It is of interest to note that, in cell-line models, the activation of PI3K in Langerhans cells was associated with an impaired immune response due to repression of genes related to immune function [62] suggesting that PI3K pathway activation may render vaccine strategies ineffective. That the immunological approach is not futile is demonstrated by the recent findings showing that vaccination with this vaccine in patients with high-grade vulvar intraepithelial neoplasia achieved a strong virus-specific T-cell immunity and was indeed associated with a complete regression of neoplastic lesions in approximately half of the patients [63]. Similar data is documented in CIN3 preneoplastic lesions using a different vaccine [36] and a phase 3 trial is ongoing with this reagent.

It is reasonable to assume that chemotherapy or targeted therapies may contribute to the successful application of immunotherapy, for instance, by providing an opportunity for effective induction. It is likely that timing of the immunotherapy in addition to chemotherapy is crucial to optimally utilize the immunostimulatory aspects of chemotherapeutics, although currently the data from treated patients are limited.

#### 3.2.6. DNA Repair Pathway

The single constant factor in cervical cancer treatment for the last 20 years is the use of platinum salts and radiotherapy, which suggests that somatic abnormalities of DNA repair enzymes may be prevalent in these tumours. To date there is little information on pretreatment DNA repair abnormalities to our knowledge. If a preexisting defect in DNA repair enzymes in cervical cancers was documented, this could lead to the use of PARP inhibitors in selected advanced and recurrent tumours. In evaluating gene expression changes as a result of chemoradiation, Klopp et al. identified modifications in genes involved in DNA repair (including DDB2, ERCC4, GADD45A, and XPC), in addition to significant changes in cell-to-cell signalling pathways such as insulin-like growth factor-1 (IGF-1), interferon, and vascular endothelial growth factor signalling [64]. Relations between pretreatment expression of components of the DNA damage recognition complex (Ku70 and Ku80 protein levels) and radiation sensitivity as well as survival have been investigated in the past [65, 66] and may need further attention.

#### 3.2.7. Multiple Pathway Activation

**Figure 1:**
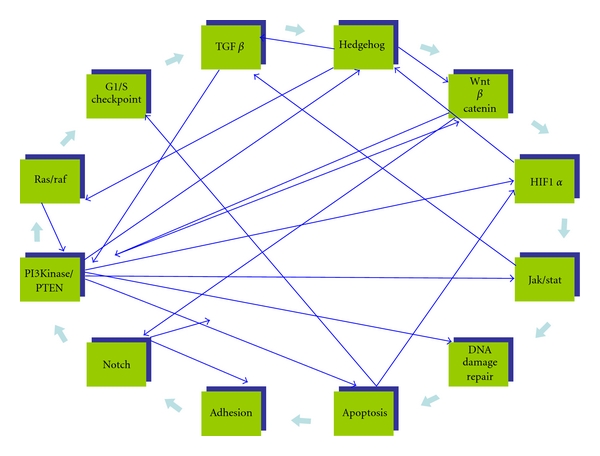
Pathway convergences and intercept nodes. This figure serves to illustrate somewhat the complexity of pathway interactions; it is not meant to be an exhaustive documentation of interactions. It is based on the references in this paper.

Based on complete exon sequencing in a variety of tumours, it was suggested by B. Vogelstein at the 2010 AACR meeting that most solid tumours have alterations in *more than one pathway*. Twelve dominant pathways were identified (Figure 1): PI3K/PTEN, ras/raf, G1/S transition, TGF*β*/smad, Wnt/beta catenin, Hedgehog, HIF-1a, Jak/stat, notch, DNA damage control, apoptosis, and adhesion. 

None of these pathways acts in isolation, and more than one pathway may need inhibiting to achieve an effective anti-tumour response. Conversely blocking one pathway may render double targeting obsolete if the second pathway is under the control of the first. There is also growing evidence for convergences between pathways.

- Convergence between Wnt/*β*-catenin and EGFR signalling has been documented by several authors. Wnt/*β*-catenin overexpression was shown to activate signalling via EGFR, while EGFR can form a complex with *β*-catenin in cancer cells thereby increasing invasion and metastasis [67]. In developmental models, Wnt and EGFR act together and they may be crosstalking differently depending on context and cell model [68]. It has been suggested in breast cancer models that interference with WNT signalling at the ligand-receptor level in combination with other targeted therapies may improve the efficiency of cancer treatments [67, 69]. Dual targeting of EGFR and angiogenesis pathways was not synergistic in early trials of cervical cancers, but the population was too small and the tolerance of full dose for either pathway inhibition was not satisfactory to draw valid conclusions. It has been suggested from breast cancer studies that lapatinib acts only on the phosphorylated form of EGFR (in the presence of wild-type ras/raf) which would mean that the number of patients potentially able to benefit represents at best 20% of the total population.
- There is ample literature data on a convergence of the PI3K/PTEN pathway with the angiogenesis pathway [11–14]. Direct inhibition of PTEN gene expression [70] via siRNA knockdown experiments was shown to cause upregulation of VEGF secretion, with increased angiogenesis, cellular proliferation and invasiveness [71]. A dual combined action within the PI3K/PTEN pathway by restoring PTEN and inhibiting PI3K synergistically suppressed glioblastoma growth in preclinical trials [72].
- Emerging data suggest that HPV may directly stimulate VEGF production through upregulation of the E6 oncoprotein [15, 16].
- Cells interact with their microenvironment. Transforming growth factor-*β* was shown to activate PI3K in HER2 overexpressing breast cancer cells, to engage stromal and endothelial cells in the tumour environment [73] and to cause Herceptin insensitivity.
- Persistent hedgehog (Hh) activation may also cooperate with other oncogenic products, such as mutated K-RAS and crosstalk with different growth factor pathways, including tyrosine kinase receptors, such as epidermal growth factor receptor (EGFR), Wnt/beta-catenin, and transforming growth factor-beta (TGF-beta)/TGF-beta receptors [74]. The hedgehog pathway may also be induced by Hif-1*α* [75].

There is recent data with dual targeting showing a sustained benefit in some patients. In metastatic head and neck tumours treated by erlotinib in association with bevacizumab, complete responses were associated with expression of putative targets in pretreatment tumour tissue [76]. But these early data must be viewed with caution due to the possibility of negative interactions as was suspected for EGFR inhibitors and angiogenesis and multi-TKI targeting, [59, 60] even though the population was not screened for target expression.

### 3.3. Future Strategies to Achieve a “Best Fit” between Optimal Clinical Information with Minimal Patient Exposure?

#### 3.3.1. Definition of Molecular Markers of Pathway Activation and of Biomarkers Predictive of Response

Several areas of investigations are currently ongoing in many institutions with the aim of precisely defining the biological target for a given drug or drug combination.

**Genetic Mutations**

There is little available data on genetic mutations in cervical cancer specimens. While EGFR is frequently activated and constitutes a primary target, there is to our knowledge no evidence of EGFR gene mutations. The following mutations in cervical cancer samples have been registered in the Sanger database by order of frequency: STK11 (serine threonine kinase) (29/201: 14%), Hras (23/263: 9%), Kras (46/636: 7%), PIK3CA (25/255: 10%), CDKN2A (23/267: 9%); in addition loss of PTEN has been documented in 16 cases. Mutations in small numbers of a variety of other genes, CTNNB1 (8), FGFR (6) Braf (5), TP53 (3), Nras (2), RB1 (2) FBXW7 (1) BRCA2 (1), and MSH2 (1), have also been reported. The STK/LKB1 gene encodes a ubiquitously expressed serine/threonine kinase that is mutated in multiple sporadic cancers and plays a role in cell growth, cell cycle progression, metabolism, polarity and migration. It is suggested that LKB1 and its neighbouring genes are frequently deregulated in primary cervical cancers and that multiple fusion transcripts are generated which may be driven by the c19orf26 promoter [77].

None of these mutations is predominant in a large fraction of the population. If, hypothetically, the presence of any one of these mutations was exclusive and if any mutation was sufficient to circumvent EGFR signalling, then EGFR inhibition might, at worst, be inoperative in 50% of patients. This is unlikely to be the case, but strategies to diagnose and overcome resistance to membrane receptor targeting are under exploration. In breast cancer cells, the lack of a response to membrane receptor inhibition was related to persistent downstream activations of PI3K (pAKT), mTOR (pS6K) and MAPK (pERK), (San Antonio Breast Cancer Conference 2010: O'Brien A: P4-01-06). The synergistic therapeutic effect of a downstream inhibitor (Rapamycin) of PI3K/AKT together with membrane receptor inhibition has been documented in preclinical assessments, and these are now tested in early clinical trials (San Antonio Breast Cancer Conference 2010: Abu-Khalaf: A: P3-14-17). Similarly, the combined use of src and MAPK inhibitors was shown to be capable of overcoming resistance to membrane receptor inhibition in selected patients (San Antonio Breast Cancer Conference 2010: Jegg A: P3-14-14).

For practical purposes, to make valid clinical decisions in the future, we need solid biomarkers relevant for each gain or loss of function at each step of the signalling network. Computer-assisted calculations of the major signalling pathways, and also of the “weaknesses” in the signalling network might, guide us to double-target selected pathways and increase treatment efficacy by synthetic lethality.

**Activated Phosphoprotein Status by RPPA (Reverse Phase Protein Array)**

RPPA consists of analysing microdeposits of protein lysates on a nitrocellulose coated slide. Up to a thousand samples can be processed in a single analysis, allowing excellent control of variability between patients. The minimal amount of material needed is 5 ng of protein sample. RPPA allows specific assessment of the activation (by phosphorylation) of tyrosine kinase pathways as well as angiogenesis, apoptosis, adhesion, the NFkB pathway, and the Jak/Stat pathway. Results are based on a comparative analysis using both a specific validated antibody for the protein of interest and, in parallel, antibody for the phosphorylated form of the protein. Valuable innovative information on activation of signalling pathways in the patients' tumours, not only prior to treatment but also according to their response to therapy, can be obtained, allowing to “fingerprint” the therapeutic effect and to establish robust predictive markers for treatment response.

#### 3.3.2. Therapies according to Tumour-Specific Alterations

A great number of chemical and biological reagents are in preclinical and clinical testing in multiple drug companies and the majority of these drugs have been designed to show activity in one of B Vogelstein's (Figure 1) 12 major pathways. 

Many of the drugs in clinical testing (Table 2) that show a precisely documented activity on well-defined cell lines and a sufficiently good tolerability in early clinical assessments may not prove of sufficient clinical interest as single agents and might risk being prematurely terminated from development. 

Drug development is costly, and there is a growing trend to use intelligent drug combinations on molecularly defined tumours. There is increasing data showing positive results of associations between chemotherapy and Parp inhibitors in DNA-repair-deficient cancers. Dual targeting by mTOR inhibitors and gefitinib in lung cancer as well as the demonstration of preclinical synergy between erlotinib and metformin in cell lines through a robust reduction of biomarkers represented by phosphorylated EGFR, AKT, ERK and S6 may pave the way for other drug associations (San Antonio Breast Cancer Conference 2010 Lau: A: PD03-05).

The choice of reagents in cervical cancers still depends on the definition of specifically activated pathway deregulation which needs further assessments. Genetic or protein biomarkers—tested before and after treatment—are crucially needed to allow prediction of activity for each individual pathway abnormality and prepare the way for multiple targeting in the future. Rigorous validation tests and interlaboratory controls are needed for most of the IHC assays on patient samples as there may be much variability as a function of sample preparation and of antibody provenance. PTEN loss has been shown to be associated with downstream pathway activation and a recent publication comparing technical aspects and demonstrating the availability of a robust PTEN assay [93] merits consideration.

Xenografts of cervical cancers are available at our centre and will allow testing of single and dual targeting of innovative therapies after extensive characterization to be done in parallel with the samples of tumours from the Cetuxicol trial.

Of upmost importance will be the clinical monitoring of activity. Early NMR or Pet scan evaluation (with RGD fluciclatide) is slikely to be helpful, but foremost cervical cancers can be biopsied before and after therapeutic intervention and the loss of activation in a given pathway should be an ideal early endpoint in future clinical trials. 

#### 3.3.3. Clinical Trial Design for Targeting Biological Pathways

Gefitinib, an anti-EGFR reagent, has been assessed in association with chemotherapy in large phase III trial designs with little or no demonstration of clinical benefit (INTACT 1&2 and others) while the same reagents, if given in selected patients with specified molecular abnormalities in their tumours, did show a major therapeutic impact [94]. In lung cancer patients, response rates of 72% could thus be demonstrated in the presence of the predictive exon 19 deletions or L858R mutations as compared to 10% in patients with wild-type EGFR. 

A classical randomized trial design is ideal *if *the biological target is well defined and present in the majority of the patient population, which *appears to be* the case for EGFR pathway activation or angiogenesis in cervical cancer. This design is problematic if there is a great number of interfering parameters (downstream activating mutations) rendering membrane receptor inhibition inoperative or if the targets can come in different states (activated, overexpressed, and mutated) and if different mutations may mean different response patterns. In cervical cancer a number of possible downstream genetic mutations are reported in the Sanger database. The absence of a response to EGFR targeting in the presence of activating ras or raf mutations has been shown in colon cancers [95–97]. If each downstream mutation prevents a clinical response via EGFR targeted inhibition, EGFR inhibitors are unlikely to make a major breakthrough. However the data that are available are from trials including only patients with advanced or relapsed tumors who are likely to harbor more than one molecular abnormality. 

Questions as to the choice of one or more targeted drugs in the presence of a complex set of alterations can only be addressed as we gain experience with molecular targeting in single settings (phase I) and in the presence of validated and robust markers of activity (phase II with biological endpoints). Vogelsteins' suggestion at the 2010 AACR meeting was to target pathways rather than individual molecules. At this stage the systematic molecular analysis of tumors by pathways is in its infancy, as is the adptive trial design necessary for personalized medicine. A promising methodological design for such a trial could be similar to that of the “BATTLE” trial in lung cancer presented by Kim at the 2010 AACR meeting [98]. The strategy was to randomize into 4 different treatments in a first instance, to be followed by an adaptative design which allowed higher recruitment into the treatment arms that had given best results in the first leg of the clinical trial.

## 4. Conclusion and Perspectives

Advanced cervical cancer remains a public health issue despite the availability of preventive vaccines and population-based screening. Preventive vaccines have become available a few years ago, but they target a very young teenage patient population with a delayed impact on cervical cancer due to the peak of cervical cancer incidence at ages 45–55. Furthermore, screening and vaccines are not widely available in the countries which are most in need, while the vaccine acceptance is now being challenged in some richer Western European countries.

Cervical cancer is accessible to repeated biopsies which make the screening for a series of molecular abnormalities before and after a strategic intervention possible. Treatment strategies can be designed according to specific molecular profiles. We do presently not know which specific gene mutations are associated with which pathway activation. We do not know which EGFR alterations or which EGFR/ras/raf/PI3K pathway alterations correlate with cetuximab activity or inefficacy, but the presence of a functional receptor in the absence of a downstream constitutive activation may prove to be a minimal requirement. The EGFR PI3kinase pathway deregulations certainly need further studies as do cyclins and cyclin-dependant kinases.

Castellino and Durden [99] put forward a hypothesis according to which, instead of inhibiting one single cell surface receptor, such as VEGFR2 with bevacizumab (Avastin), thereby leaving a significant number of receptors free to pulse angiogenic signals, a more effective strategy may be to regulate signalling through targeting an *intercept node* where cell surface receptor signals *converge* (Figure 1) to transmit important signalling events within the cell and thereby bring a coordinate control over multiple pathways. These intercept nodes need defining though with pre- and post therapeutic molecular “footprinting” following single and double targeting agents. 

There is a need to coordinate efforts within national and international groups to allow patient recruitment in a reasonable time frame, the added benefit being that multinational trials will also favour harmonization in standards of care.

## Figures and Tables

**Table 1:** Ongoing trials with targeted agents.

	Products	Trial
1	CDDP-Paclitaxel versus Topotecan-Paclitaxel ± bevacizumab	GOG 204
2	Poly tki (pazopanib) versus EGFR/HER2 dual inhibitor (lapatinib)	VEG 105281
3	VEGF and FGF2 inhibitor: Brivanib	227G
4	EGFR inhibitor erlotinib	GOG 227 D
5	EGFR-targeted antibody cetuximab	GOG 227 E
6	EGFR inhibition by cetuximab + CDDP	GOG 76DD
7	Vaccine: listeriolysin O E7 fusion peptide: ADXS11:	GOG 285
8	G1 checkpoint modulation, MK1775*, inhibitor of Wee1 kinase activity	GOG 265
9	Treatment with oncolytic viruses: PV701**	PV701
10	Farletuzumab and folate receptor antibody coupled to vinca drug***	

*Inhibitor of Wee 1 kinase activity promotes apoptosis when p53 is null.

**Targets defect in interferon pathway (pathway disabled by HPV E6 and E7).

***Folate receptor expressed in 1/3 of (most aggressive) cervical cancers.

**Table 2:** Potential biomarkers according to pathway.

Pathway activation	Reagents	Diagnostic or predictive biomarkers under scrutiny
PI3K/PTEN*	PI3K inhibitors: PI103, BGT226 mTor inhibitors: temsirolimus, everolimus, ridaforolimas AKT inhibitors: perifosine, GSK690693 and MEK inhibitors	? pERK

Ras/raf pathway**	Ras inhibitors: tipifanib, lonafnaib Raf inhibitor: sorafenib,PLX4032 MAPK inhibitor: AZD6244,XL518	tbd

HIF-1*α* (angiogenesis)	Bevacizumab, VEG105281, Brivanib, Sunitinib, AZD2171 HIF1*α* inhibitor: Adaphostin	Tbd

G1 checkpoint	Checkpoint inhibitor: MK1775*** ****? Retinoic acid and Topotecan	? p53, p63, p73 ? Wee-1, Myt1, ChK1, CDC2

Apoptosis*****	? Anti HPV vaccine indicated here	? p53, p63, p73; ? Inactivation of p15 ± RB,bcl-xl

DNArepair: homologous recombination deficient	PARP inhibitors	? DNA damage control deficiency/Homologous recombination BRCA2 mutation (rare)

Jak/stat	? PV-701 (oncolytic virus) targets interferon pathway defects which are induced by HPV E6 and E7	? IL8 (−) ? B cell signature (+) ? T regulatory cells (−)

TGF *β*	?	?IL10, TGF *β*

Adhesion	?	Tbd

Hedgehog	BMS-83392, IPI 926, LDE225, LEQ 506, GDC-0449, PF 04449913, TAK 441,	Tbd

Wnt/*β* Catenin******	Wnt1, Wnt2: neutralizing antibodies AINS, Vit D PKF115-584, PKF222-815, CPG049090 NSC668036 Oncolytic adenoviruses modified to target Tcf-*β* Catenin Antisense Avi-4126 targets end product *c-myc *	

Notch	Neutralizing Antibodies against ligand	Tbd, ? AKT

—	Farletuzumab	Folate receptor

—	Folate receptor coupled to vinca drug	Folate receptor

Neighboring cells	Active on tumour micro-environment	Tbd

Multi pathway inhibitors	Pazopanib******	Tbd

*PIK3 amplification or mutational activation or PTEN loss. Activation of the PIK3/AKT/mTOR pathway was associated with a worse prognosis and chemoresistance in cervical cancers [78].

“?” means not validated.

**Ras/raf pathway alterations in colon cancer render these tumours resistant to an EGFR inhibitor. Ras inhibitors such as Tipifanib or lonafanib did not show relevant clinical activity in phase II and III studies in various tumour types [79] and may be worth assessing again in single and dual targeting in selected preclinical models. ***E6 mediated inactivation of p53 upregulates VEGF and angiogenesis. ****Retinoic acid and topotecan may be useful in tumours with checkpoint activation [80]. *****There is evidence that human papillomavirus infection enhances phosphorylation of retinoblastoma protein and decreases apoptosis in a particularly aggressive type of squamous cell carcinoma of the uterine cervix [20–23]. ******Wnt/*β* Catenin inhibition: by antibodies [81–83], AINS, [84–86], VitD3 [87], or small molecules [88–90] or oncolytic viruses [91]. Also antisense, molecule to end product c-myce [92].
